# Bioinspired Multistimuli‐Induced Synergistic Changes in Color and Shape of Hydrogel and Actuator Based on Fluorescent Microgels

**DOI:** 10.1002/advs.202304776

**Published:** 2023-11-27

**Authors:** Dongdong Lu, Qing Lian, Mingning Zhu

**Affiliations:** ^1^ School of Physical Sciences Great Bay University Dongguan 523808 P. R. China; ^2^ Derpartment of Materials Science and Engineering Southern University of Science and Technology Shenzhen 518055 P. R. China; ^3^ School of Biomedical Engineering Guangdong Medical University Dongguan 523808 P. R. China

**Keywords:** biomimetic, fluorescence, hydrogels, microgels, multiresponsive

## Abstract

Fluorescent hydrogels have emerged as one of the most promising candidates for developing biomimetic materials and artificial intelligence owing to their unique fluorescence and responsive properties. However, it is still challenging to fabricate hydrogel that exhibits synergistic changes in fluorescence color and shape in response to multistimulus via a simple method. Herein, blue‐ and orange‐emitting fluorescent microgels (MGs) both are designed and synthesized with pH‐, thermal‐, and cationic‐sensitivity via one‐step polymerization, respectively. The two fluorescent MGs are incorporated into transparent doubly crosslinked microgel (DX MG) hydrogels with a preset ratio. The DX MG hydrogels can tune the fluorescent color accompanied by size variation via subjecting to external multistimulus. Thus, DX MG hydrogels can be exploited for multiresponsive fluorescent bilayer actuators. The actuators can undergo complex shape deformation and color changes. Inspired by natural organisms, an artificial morning glory with color and size changes are showcased in response to buffer solutions of different pH values. Besides, an intelligent skin hydrogel, imitating natural calotes versicolor, by assembling four layers of DX MG with different ratios of MGs, is tailored. This work serves as an inspiration for the design and fabrication of novel biomimetic smart materials with synergistic functions.

## Graphical abstract

Bioinspired responsive and color‐switchable doubly‐crosslinked microgel hydrogels are constructed by blue‐ and orange‐emitting fluorescent microgels. The smart hydrogels can tune the fluorescent color accompanied by size variation via subjecting to external multistimulus. The gels can be developed to fluorescent actuators, artificial morning glory, and biomimetic skin of calotes versicolor.

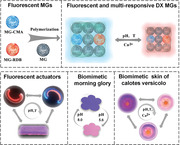

## 1 Introduction

Many animals and plants in nature showcase a wide range of morphological and color adaptations in response to shifts in their surroundings, a phenomenon pivotal for executing intricate tasks such as predator avoidance, communication, and reproduction.^[^ ^1^ ^]^ For example, chameleons are capable of modifying their body color to blend into their habitat for camouflage or hunting.^[^ ^2^ ^]^ Octopuses, known as the “chameleons of the sea,” possess astonishing color‐changing abilities. They can rapidly alter the color of their skin to match their surroundings, and also display color shifts indicative of their emotional states. Their skin is usually brownish‐purple but turns reddish‐brown when angry, grayish‐white when fearful, and sometimes even brown.^[^ ^3^ ^]^ These fascinating color changes emerges from intricate movements/deformations and transformations of optical properties, including reflection, scattering, absorption, refraction, and even luminescence, triggered by environmental stimuli.^[^ ^2^, ^4^ ^]^ Quisqualis indica also orchestrates its own captivating transformation. Initially unfolding as white blossoms during twilight, they transition to pink the following day, eventually culminating in a vibrant red hue.^[^ ^5^ ^]^ This behavior can facilitate pollination because the white flowers reflect light at night for attracting moths, while the vibrant red color during the day appeals to bees and butterflies.^[^ ^6^ ^]^ It is due to the volatility of anthocyanin in the petals, which is influenced by environmental factors such as acidity, alkalinity, and temperature. These exciting phenomena inspired scientists to create stand‐alone intelligent artificial materials,^[^ ^7^ ^]^ particularly those with integrated color‐tuning capabilities in soft materials, resulting in intelligent soft robotics, such as camouflage robots, exhibiting multifunctional synergies and adapting to the environment in time.^[^ ^8^ ^]^

In recent years, smart fluorescent hydrogels (SFHs) have gained significant attention for their applications in fabricating biomimetic intelligent materials.^[^ ^9^ ^]^ These hydrogels offer a unique combination of hydrogel properties^[^ ^10^ ^]^ and fluorescent polymers,^[^ ^11^ ^]^ allowing them to exhibit color and shape changes. The most popular approach involves the physical doping or covalent grafting of two or more responsive fluorophores (like luminescent proteins,^[^ ^12^ ^]^ organic fluorophores,^[^ ^13^ ^]^ and fluorescent nanoparticles,^[^ ^14^ ^]^ lanthanide complexes^[^ ^15^ ^]^) into a single stimuli‐responsive gel matrix.^[^ ^16^ ^]^ Those showed the responsiveness to external stimuli (e.g., light, temperature, pH, and solvent) by accompanying the color changes.^[^ ^17^ ^]^ Recently, Chen and co‐workers developed anisotropic bilayer hydrogel actuators with an on–off switchable fluorescent color‐changing function.^[^ ^18^ ^]^ They combined a thermosensitive graphene oxide‐poly(*N*‐isopropylacrylamide) hydrogel layer with a pH‐sensitive perylene bisimide‐functionalized hyperbranched polyethylenimine hydrogel layer via supramolecular assembly. Tang and co‐workers designed a bilayer hydrogel strategy by utilizing an aggregation‐induced emission luminogen to fabricate hydrogels.^[^ ^19^ ^]^ By adjusting the pH values, these actuators exhibit fluorescence color changes, deformation, and brightness alterations.

However, those approaches raise several issues. First, fluorescence deficiencies (including limited tunability of fluorescence color, poor sensitivity in color‐changing, and difficult‐to‐interpret mechanisms) will occur due to the unpredictable and complex photophysical processes (e.g., resonance energy transfer and excimer emission) of randomly organized different fluorophores.^[^ ^20^ ^]^ Another problem arises concerning the harmonization of color, luminosity, and form within SFHs. Although there have been reported deformable hydrogels exhibiting independent stimulus‐driven changes in fluorescence color and shape,^[^ ^19^, ^22^ ^]^ these changes rely on distinct constituents within the hydrogel or disparate triggering stimuli. Thus, achieving responsive hydrogels that can responsively and synergistically alter both fluorescence color and shape in response to stimuli remains a challenge. Additionally, the majority of existing SFHs are designed to react to singular external stimuli, which inadequately simulates the complex natural environment.^[^ ^19^, ^23^ ^]^ Developing multiresponsive biomimetic materials becomes imperative to overcome this limitation. Besides, the luminescent substances without interacting with the polymeric network will inevitably leak from hydrogel matrix. These leaching precipitates erratic color transformations induced by stimuli. The current common methods to incorporate hydrophobic fluorophores into the hydrogen network involve the usage of environmentally unfriendly and biologically toxic organic solvents.^[^ ^9b^ ^]^ This undoubtedly imposes substantial constraints on the bionic materials applications of SFHs.

**Scheme 1:**
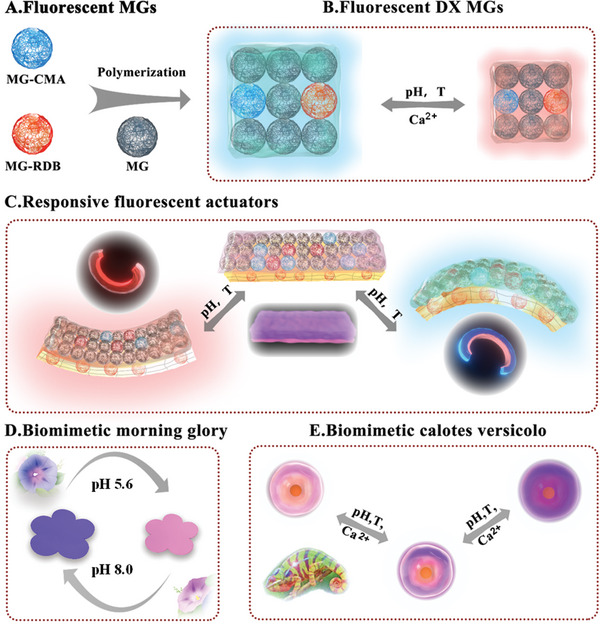
Schematic illustration of A) MG‐CMA and MG‐RDB, which are constructed to B) fluorescent and multiresponsive DX MGs hydrogels. C) Simultaneous changes in fluorescent color and shape of a DX MGs‐based bilayer hydrogel actuator and D) biomimetic flower of morning glory with color and size variation in response to acidity/alkalinity stimuli. E) Biomimetic skin of calotes versicolo, constructed from four layers of DX MG with different ratios of MG‐CMA and MG‐RDB, with multicolor changing induced by pH, temperature, and cationic solution.

Herein, to overcome those issues discussed above, we designed and fabricated synergistic changes in fluorescence color and size of hydrogels under multiple stimuli (pH, temperature, and cation). We achieved this by utilizing two different types of fluorescent microgels (MGs) with blue and orange fluorescence, denoted as MG‐CMA (where CMA is Coumarin) and MG‐RDB (with RDB standing for Rhodamine B), respectively (**Scheme** **1**A). Fluorophore monomers were copolymerized into MGs. Both MGs showed pH‐, thermal‐, and cationic‐sensitivity concomitant with fluorescence behavior and diameter variation. The fluorescence variation arises from pH sensitive, π–π* overlap of the neighboring fluorophore and innerfilter effect predictably, without other complex photophysical processes. The water swellable MG‐CMA and MG‐RDB were incorporated into doubly crosslinked microgel (DX MG) hydrogels to form luminescent hydrogels without using environmentally unfriendly or biologically toxic organic solvents (Scheme 1B). Hence, our hydrogels were not cytotoxic. In contrast to conventional techniques involving the direct copolymerization or grafting of fluorophores into the hydrogel matrix, the fluorescent MGs ensure the uniform distribution of microlocalized high‐concentration of fluorophores within the hydrogel matrix. Combined with DX MGs hydrogels wherein vinyl‐functionalized and multiresponsive MGs serve as building blocks, this strategy achieves synergistic alterations in both fluorescence color and swelling characteristics triggered by multistimuli. It was also shown to enable the construction of cytocompatibility and responsive fluorescent hydrogel actuators (Scheme 1C). To demonstrate the proof of concept, we created an artificial morning glory that exhibited color and size variation in response to acidity/alkalinity stimuli from buffer solution (Scheme 1D). In addition, we also developed intelligent skin hydrogels by assembling the four layers of DX MG with different ratios of MG‐CMA and MG‐RDB. It displays simultaneous changes in size and multicolor, similar to that of the natural calotes versicolor (Scheme 1E). Our distinctive approach offers a promising alternative for creating stimuli‐responsive fluorescent hydrogels with multiple properties, overcoming the constraints inherent in previously reported SFH.

## 2 Result and Discussion

### 2.1 Fluorescent and Multiresponsive Microgels

**Figure 1:**
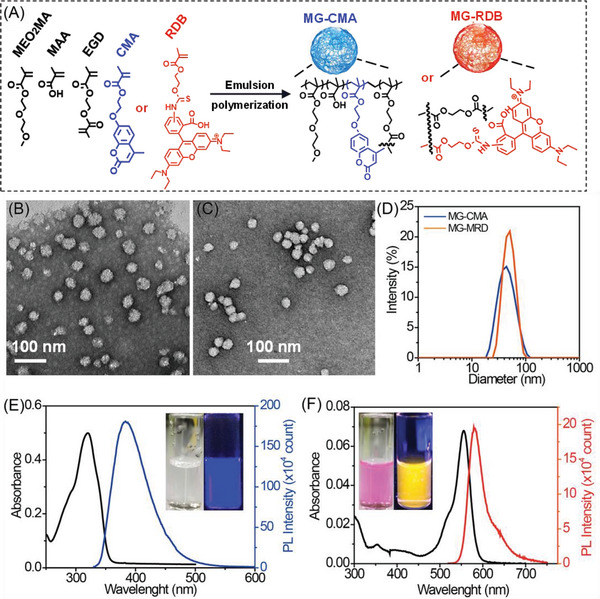
A) Schematic MG‐CMA and MG‐RDB synthesis. TEM images for B) MG‐CMA and C) MG‐RDB, D) DLS distribution of as made MGs, E) UV–vis and F) photoluminescence (PL) spectra for as made MG‐CMA and MG‐RDB. The pH value for as made MGs dispersion is 4.5. The inset shows the dispersions at white light (left) and UV light with 302 nm. The diameter of glass vials was 10 mm. (E,F) The concentration of MGs dispersion is 0.01 wt%. The data were obtained at 25 °C.

Both CMA and RDB contain a methacrylate moiety, rendering them amenable to integration within polymers via radical polymerization. MG‐CMA and MG‐RDB can be synthesized via emulsion polymerization (**Figure** **1**A). Transmission electron miscroscope (TEM) images (Figure 1B,C) revealed that the MG‐CMA and MG‐RDB had a number‐average diameter of 30 and 37 nm, respectively (Figure S1, Table S2, Supporting Information). Deswollen (collapsed) MG‐CMA and MG‐RDB, as measured by dynamic light scattering (DLS) at pH 4.5 and 25 °C, had an average diameter of 40 and 48 nm, respectively (Figure 1D; Table S2, Supporting Information), and were negatively charged as shown by electrophoretic mobility data (Figure S2, Supporting Information). Potentiometric titration data (Figure S3, Supporting Information) were used to determine the methacrylic acid (MAA) contents and these values were 26.7 and 27.6 mol% for MG‐CMA and MG‐RDB, respectively. The particles contained CMA (6.35 mol%) and RDB (0.25 mol%) as determined by UV–vis spectroscopy and the Beer–Lambert law (see Figure 1E,F; Figure S4, Supporting Information). The MG‐CMA and MG‐RDB exhibited photoluminescence (PL) emission spectra over the wavelength range of 340–530 nm with a maximum at 383 and 550 to 700 nm with a maximum at 581 nm, respectively (Figure 1E,F). When illuminated at 302 nm, the MG dispersion emitted dark blue and bright orange fluorescence (inset of Figure 1E,F).

**Figure 2:**
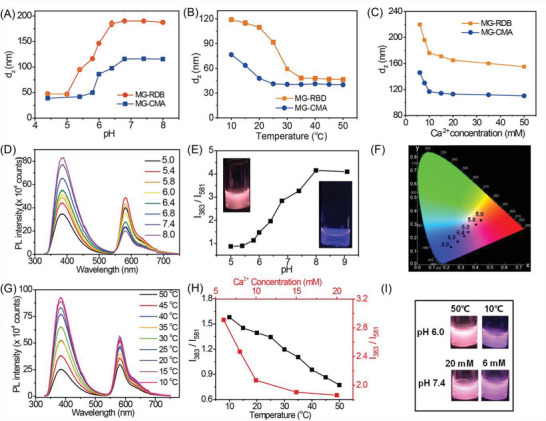
Variation of A) *z*‐average diameter (*d_z_*) with pH, B) temperature at pH 6.0, C) different concentration of CaCl_2_ at pH 7.4 for MGs dispersion. The concentration for MGs for (A)–(C) is 0.01%. D) Photoluminescence (PL) spectra, E) the ratio change at *I*_383_/*I*_581_, and F) CIE diagram of MGs dispersion mixture with different pH values. G) PL spectra of MGs dispersion mixture with different temperatures at pH 6.0. The ratio changes at *I*_383_/*I*_581_ (G) and I) photos at UV lamp with 302 nm of MGs dispersion mixture with different temperatures at pH 6.0 and different CaCl_2_ concentrations at pH 7.4 and 25 °C. (D–I) The MGs dispersion mixture contains 0.07 wt% MG‐CMA and 0.07 wt% MG‐RDB. (D–F) The data were obtained at 25 °C. (E,I) The diameter of glass vials was 10 mm.

The MGs were multiresponsive (pH, temperature, and cation) due to the presence of poly(methacrylic acid) (PMAA) and poly(2‐(2‐methoxyethoxy)ethyl methacrylate) (PMEO_2_MA) segments. The effects of multistimuli on MGs size were investigated using DLS. The variations of *z*‐average size (*d_z_*) with pH for the MG‐CMA and MG‐RDB dispersions measured at 25 °C (**Figure** **2**A). The *d_z_* values increased as the pH approached the respective apparent p*K*_a_ values (6.0 and 5.5 for MG‐CMA and MG‐RDB, respectively) because the deprotonation of RCOOH group from PMAA and electrostatic repulsion of RCOO^−^ groups between polymer chain. Zeta potential (*ζ*) data measured via electrophoretic mobility for the MGs over the pH range of 4.5 to 7.4 at 25 °C implied that the negative charge density near the MG surface increased in magnitude with increasing pH (Figure S2, Supporting Information). As the temperature increased at pH 6.0, the MGs deswelled due to thermally triggered disruption of hydrogen bonding between water and the PMEO_2_MA, which causes hydrophobic, attractive interactions between polymer chains to dominate^[^ ^23^ ^]^ (Figure 2B). It can be seemed the discernible distinctions existed in the pH‐/temperature‐induced collapsing/swelling behavior of MG‐CMA and MG‐RDB. It is attributed to variations of hydrophobicity and crosslinking density within the polymer network between them. The relative smaller content of PMAA and substantial presence of CMA lead to more hydrophobic polymer network in MG‐CMA than that of MG‐RDB. Besides, inevitable photodimerization of neighboring coumarins, engenders a crosslinked polymer network.^[^ ^24^ ^]^ Hence, the hydrophobic polymer chain and crosslinking point inhibit the pH‐/temperature‐ triggered MG swelling and decrease the VPTTs values.

We selected CaCl_2_ to investigate the ionic strength effect on the MGs at pH 7.4 because the ionic sensitivity of MGs is the result from RCOO^−^ group and RCOOH groups are complete deprotonation at this pH value. The *d_z_* values (Figure 2C) decreased significantly from 147 to 116 nm and 220 to 173 nm for MG‐CMA and MG‐RDB, respectively, when the aqueous CaCl_2_ concentration increased from 0.006 to 0.010 m. The *d_z_* values remained low but still swollen above their fully collapsed diameters as further increases in the CaCl_2_ concentration. It confirms the neighboring RCOO^−^ groups are not able to approach each other sufficiently closely to enable formation of Ca(─COO^−^)_2_ crosslinks. Hence, the collapse behavior of the MGs is due to electrostatic screening,^[^ ^25^ ^]^ which arises from electric fields generated by the Ca^2+^ tends to neutralize that from negative charges of RCOO^−^ groups and reducing the effective strength of electrostatic interactions.

The PL spectra obtained from the MG‐CMA and MG‐RDB dispersion mixture (0.07 wt% for both two MGs, respectively) were also sensitive to pH, temperature, and cation. As the pH increased from 5.5 to 9.8, the maximum PL intensity of MG‐CMA (*I*_383_) increase while that of MG‐RDB (*I*_581_) decreased (Figure 2D). In contrast to dissolve MCA and RDB in solvents, the copolymerization of MCA and RDB within the MG matrix readily engenders localized high concentrations of fluorophores. This heterogeneous distribution renders our MG system susceptible to invoking an inner filter effect. The inner filter effect refers to the phenomenon that the absorbance of the excitation light by the sample leads to a reduction of fluorescence intensity at high fluorophores concentration.^[^ ^26^ ^]^ During particles swelling at elevated pH values, the increase in distance between polymerized CMA within the polymeric network that leads to a decrease localized fluorophores concentration and reduction of innerfilter effect, which ultimately resulting in an increase *I*_383_ for MG‐CMA. As for MG‐RDB, in addition to this reason, the spiro‐ring opening process of RDB induced by H^+^ leads to high fluorescence of MG‐RDB. Hence, the *I*_383_/*I*_581_ ratio increased (Figure 2E) with the change of CIE coordinates from (0.41, 0.30) to (0.24, 0.14) (Figure 2F) as pH improved. The dispersion mixture changed from pink to violet when illuminated with UV light (Figure 2E, inset). The individual MGs dispersion also showed a similar trend (Figure S5, Supporting Information).

The PL spectrum of dispersion mixture was dependent on temperature. The fluorescence intensity of dispersion gradually decreases with the increase in temperature at pH 6.0 (Figure 2G). These observations are attributed to both the improvement of the innerfilter effect from MGs collapsed and heat‐facilitated nonradiative deactivation processes.^[^ ^27^ ^]^ The decrease in fluorescence intensity for MG‐RDB was not as strong as that for MG‐CMA. We considered it as a different amount of fluorescence monomer polymerized in MGs. The amount of RDB was much smaller than CMA in their MGs (Table S2, Supporting Information). The effect of MGs deswollen induced inner‐filter for MG‐RDB was more slightly than that for MG‐CMA. Hence, the *I*_383_/*I*_581_ ratio of two MG dispersion mixture decreased (Figure 2H) and the color changed from purple to pink (Figure 2I) as temperature raising. It was observed that increasing the concentration of MG‐CMA and MG‐RDB dispersions caused a pronounced redshift of *λ*_max_ and also changed in *I*_max_ (Figure S6, Supporting Information). The redshift is due to π–π* overlap of neighboring fluorophore. The *I*_max_ increased first is due to fluorophore concentration improvement and then decreased due to the inner filter effect. The concentration of two MGs at their respective biggest values of *I*_max_ were different (0.02 and 0.25 wt% for MG‐CMA and MG‐RDB, respectively) owing to the difference in fluorophore monomer polymerized. The results confirmed the different variation in size‐induced innerfilter effect derived by temperature upon the two MGs at pH 6.0. At pH 7.4, even though the size was independent on both two MGs, the *I*_max_ values decreased as temperature increase due to heat‐facilitated nonradiative deactivation processes. However, the *I*_383_/*I*_581_ values kept stable throughout all temperatures (Figure S7, Supporting Information). It proves again that, in terms of thermally induced alterations in fluorescent behavior, it is size but not nonradiative deactivation that affects the *I*_383_/*I*_581_ ratio at pH 6.0.

The *I*_max_ values and *I*_383_/*I*_581_ ratio decreased for the MGs mixture with an increase in CaCl_2_ concentration due to MGs collapsed (Figure 2H; Figure S8, Supporting Information). The color also changed (Figure 2I). The lowest energy transition is localized to the xanthene system of the rhodamine compound,^[^ ^28^ ^]^ indicating Ca^2+^ cannot affect the fluorescence intensity. Thus, the change in fluorescence behavior with Ca^2+^ concentration is solely due to the variation in the size of the MGs. Interestingly, the decreasing trends of *I*_max_ values and *I*_383_/*I*_581_ ratio (Figure 2H; Figure S8, Supporting Information) were synchronous with the *d_z_* changes (Figure 2C) with both of them sharply decreased before the 10 mm threshold of the introduced electrolyte, and remained stable thereafter. It proves the size decrease induced by the presence of electrolyte solutions engenders the innerfilter effect.

Förster resonance energy transfer (FRET) is important for affecting the fluorescent behavior of two or more fluorophores in one system.^[^ ^29^ ^]^ Herein, we considered the size variation of two MG mixture did not lead to FRET. FRET only occurs when the emission and absorption of two or more dyes overlap and the average separation between the dyes is less than 10 nm. The average separation between particle centers (*L*) can be calculated using the equation *L* = *N*_p_^1/3^, where *N*_p_ is the number density of particles.^[^ ^30^ ^]^ Using the value for *d*_TEM_ (33 nm) (Figure S9, Supporting Information), values of *L* for mixture MGs dispersion studied here (0.07 wt%) is calculated as 130 nm. Hence, this concentration is used to prepare fluorescent and multiresponsive hydrogels with the consideration that the FRET will not occur in this gel.

### 2.2 Multistimuli‐Induced Synergistic Changes in Color and Shape of DX MG Hydrogel

**Figure 3:**
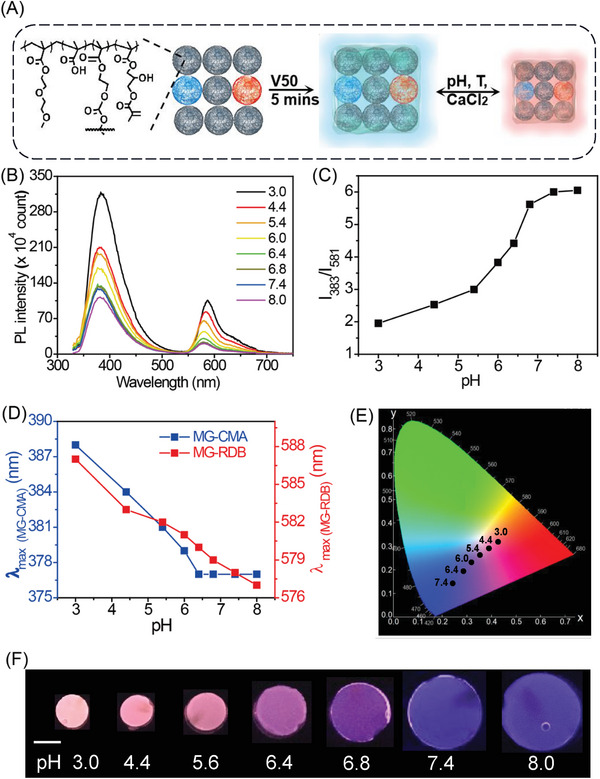
A) Depiction of DX MG‐CMA_0.07_/RDB_0.07_ hydrogels synthesis. B) PL spectra, C) the ratio values at *I*_383_/*I*_581_ and D) the λ_max_ changes for the gels after immersed in buffer solutions of different pH values. E) The CIE diagram and F) photos of gels at UV lamp with 302 nm. (F) The scale bar is 10 mm. The data were obtained at 25 °C.

We next incorporated MG‐CMA or/and MG‐RDB into OEG‐based doubly crosslinked microgel (DX MG) to form pH‐, thermal‐, and cationic‐responsive hydrogels, namely, DX MG‐CMA*_x_*/RDB*_y_*. *x* and *y* represent the concentration of MG‐CMA and MG‐RDB (wt%) in DX MG, respectively (**Figure** **3**A). The glycidyl methacrylate (GMA)‐fictionalized OEG‐based MG (GMG‐MEO) was used as building blocks. (The composition and detailed information is showed in Table S2, Supporting Information.) In contrast to conventional methods of preparing fluorescent hydrogel (by doping or copolymerizing fluorescent material within hydrogel), our constructing procedure can avoid fluorescein leakage because the dyes were copolymerizing into MGs and freedom fluorescein were washed with chloroform and dialyzed before forming covalent bonds between fluorescent MGs and GMG‐MEO. All CMA and RDB were covalently linked to the polymeric network of DX MG gels. It can undoubtedly enhance the stability of luminous performance of gels. Our MGs are water‐swellable, which can avoid the usage of environmentally and biologically harmful organic solvents. Besides, uniform distribution of microlocalized high‐concentration of fluorophores within the hydrogel matrix and the multiresponsive MGs severing as building blocks ensure synergistic alterations in both fluorescence color and swelling characteristics triggered by multistimuli. This approach can be a general strategy for constructing smart and stable fluorescent soft material with an eco‐friendly and nontoxic approach.

Both DX MG‐CMA and DX MG‐RDB were highly transparent under daylight. The emission color of DX MG hydrogel can judiciously tunable through the manipulation of the MG‐CMA and MG‐RDB ratios (Figure S10, Supporting Information). The PL spectrum revealed that the intensity of the blue band at 383 nm increased at the cost of the intensity of the red band at 581 nm upon an increase in the MG‐CMA/MG‐RDB ratio (Figure S10, Supporting Information). We selected the DX MG by constructing from the dispersion mixture of MG‐CMA/MG‐RDB (comprising 0.07 wt% of MG‐CMA and 0.07 wt% of MG‐RDB), namely DX MG‐CMA_0.07_/RDB_0.07_, to conduct the following experiment.

The pH‐responsiveness of DX MG‐CMA_0.07_/RDB_0.07_ was investigated. Those gels were immersed into a series of buffer solutions ranging pH values from 3.0 to 8.0. Fluorescence photographs (Figure 3F) and data on λ_max_ and *I*_383_/*I*_581_ (Figure 3B) demonstrated the high pH sensitivity of DX MG‐CMA_0.07_/RDB_0.07_. As the pH raised, *I*_383_/*I*_581_ underwent a significant transition from 1.95 to 6.2, before stabilizing at pH levels above 7.4 (Figure 3C). The λ_max_ blue‐shifted ≈10 nm for both CMA and RDB (Figure 3D). The swelling ratio (*Q*_DX MG_) also increased from 2.3 at pH 3.0 to 47.2 at pH 8 (Figure S13, Supporting Information). Concurrently, the fluorescent color changed from pink to dark blue, which corresponds to the change of CIE coordinates from (0.38, 0.23) to (0.22, 0.15) (Figure 3E). By contrast, when fluorescent MGs was incorporated within a nonsensitive polyacrylamide (PAAm) gel, the λ_max_ remained almost stable and *I*_383_/*I*_581_ increased by 3.2 with pH fluctuations (Figure S14, Supporting Information). We attributed the larger change of *I*_383_/*I*_581_ and redshift of λ_max_ observed for DX MG‐CMA_0.07_/RDB_0.07_ to both pH sensitivity of the fluorescent MGs and the strong π–π* overlap stemming from more pronounced MGs shrinkage due to small *Q*_DX MG_ at low pH values. The fluorescent changes induced by pH are less significant of PAAm‐MG‐CMA_0.07_/RDB_0.07_ because it is only affected by the pH sensitivity of fluorescent MGs.

**Figure 4:**
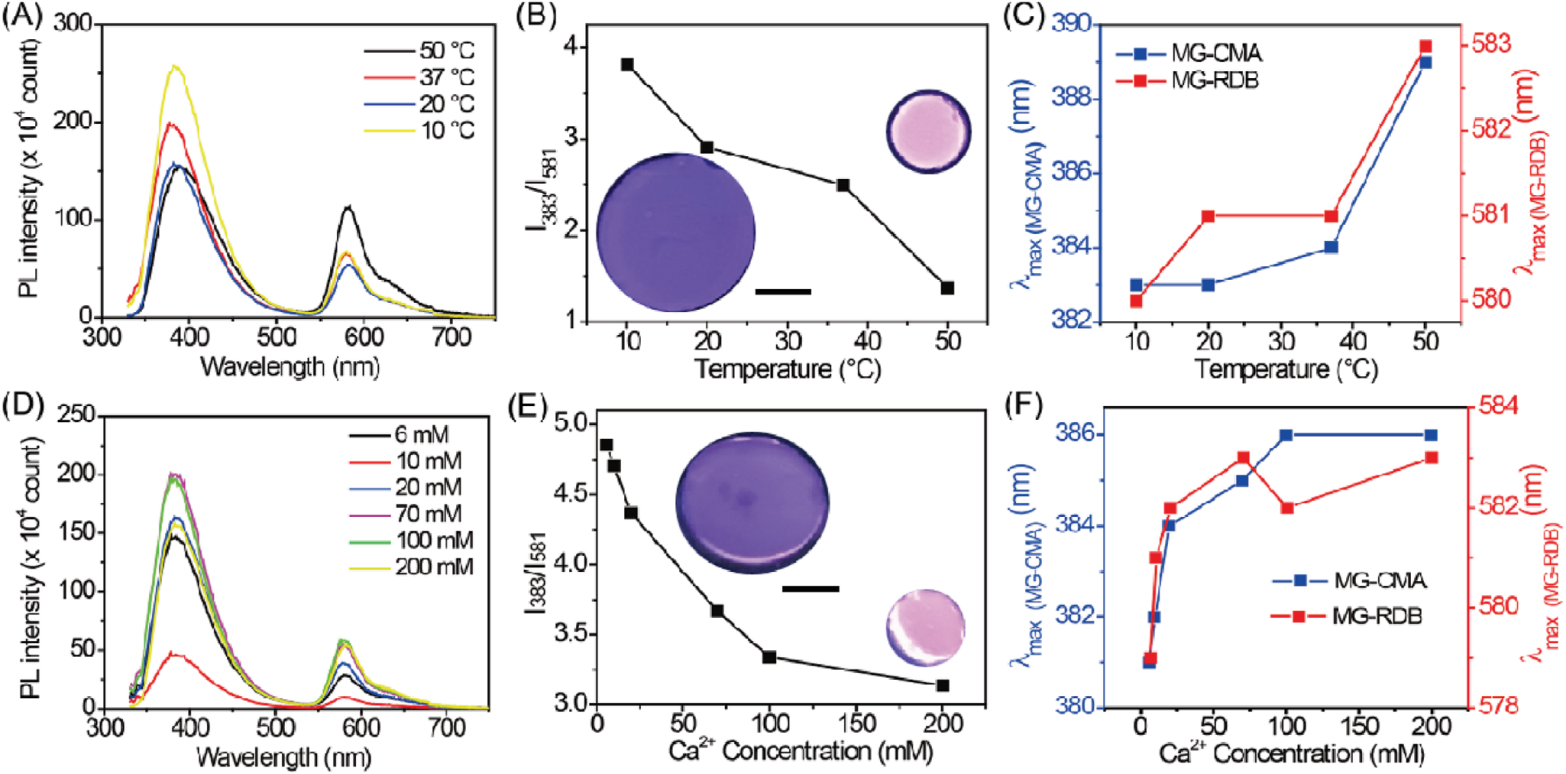
A) PL spectra of DX MG‐CMA_0.07_/RDB_0.07_ with temperature at pH 6.0 and D) varying CaCl_2_ concentration at pH 7.4. B) The ratio values at *I*_383_/*I*_581_ of gels with temperature at pH 6.0 and E) CaCl_2_ concentration at pH 7.4. C) The λ_max_ changes with temperature at pH 6.0 and F) CaCl_2_ concentration at pH 7.4. The inset images in (B) were the gel at temperatures (10 and 50 °C) and (E) at CaCl_2_ concentration (6 and 200 mm), respectively. (B,E) The scale bars are 10 mm. The data for the study of gel with CaCl_2_ concentration variation were obtained at 25 °C.

The multiresponsiveness of hydrogels is crucial to the intelligence of biomimetic materials.^[^ ^16^ ^]^ We have proved the thermal and cationic responsiveness of OEG‐based DX MGs in previous study.^[^ ^25^ ^]^ Herein, we next studied the impact of cation and temperature to the fluorescent behaviors of DX MG‐CMA_0.07_/RDB_0.07_. The *Q*_DX MG_ values decreased as the temperature increased due to the interactions change of water with the ether oxygens^[^ ^31^ ^]^ (Figure S15A, Supporting Information). The temperature‐triggered deswelling was much less significant at pH 7.4 owing to the electrostatic repulsion of RCOO^−^ group overcomes hydrophobic interactions (Figure S15A, Supporting Information). At pH 6.0, as temperature increased, *Q*_DX MG_ values decreased from 35.0 at 10 °C to 18.6 at 50 °C (Figure S15A, Supporting Information). The *I*_383_/*I*_581_ of DX MG‐CMA_0.07_/RDB_0.07_ decreased by a factor of 2.8, and λ_max_ redshift around 5 nm for MG‐CMA and 3 nm for MG‐RDB (**Figure** **4**A–C). It is important to note that the temperature can affect the ratio of *I*_383_/*I*_581_ but not λ_max_ of MG‐RDB and MG‐CMA dispersion mixture as above discussion. However, the MG‐RDB and MG‐CMA are incorporated and fixed into DX MG. When the DX MG undergoes deswelling, the physical distance of fluorescent MGs become closer and the collapsing behavior of particles was stronger within the gel than that in dispersion. It is due to polymer chains entangled and/or grafted to the MGs surface, and the strong gel network swelling (or deswelling) provides an additional force that further enlarged (or collapsed) the particles.^[^ ^32^ ^]^ The DX MG deswelling leads to stronger π–π* overlap and innerfilter effect of neighboring fluorophores groups. By contrast, even though the MGs also collapsed with increasing temperature at pH 6.0, the distance of particles did not change in dispersion and the degree of collapsed was not as evident compared to that in gel network. Hence, the temperature‐dependent of fluorescent color for DX MG‐CMA_0.07_/RDB_0.07_ is more evident with a dark blue color at 10 °C and a pink color at 50 °C (Figure 4B, inset images).

To investigate the effect of added electrolytes on the fluorescent properties and swelling behaviors of DX MG‐CMA_0.07_/RDB_0.07_, the gels were equilibrated in solutions of varying CaCl_2_ concentrations. The *Q*_DX MG_ values decreased from 32.5 to 12.8 as the concentration of CaCl_2_ increased from 6 to 200 mm (Figure S15B, Supporting Information). The gel collapsed as the ionic strength of the solutions increase due to ionic screening raised.^[^ ^25^ ^]^ Both smaller size of particles and distance between MG‐RDB and MG‐CMA lead to π–π* overlap and the innerfilter effect of fluorescent group. Hence, the *I*_383_/*I*_581_ decreased and λ_max_ redshifted for gels exposed to higher CaCl_2_ concentration (Figure 4D–F). The fluorescent color also became from purple to pink with the changes of CIE coordinates from (0.30, 0.20) to (0.37, 0.27) (Figure S16, Supporting Information).

### 2.3 Fluorescent Actuators and Biomimetic Application

**Figure 5:**
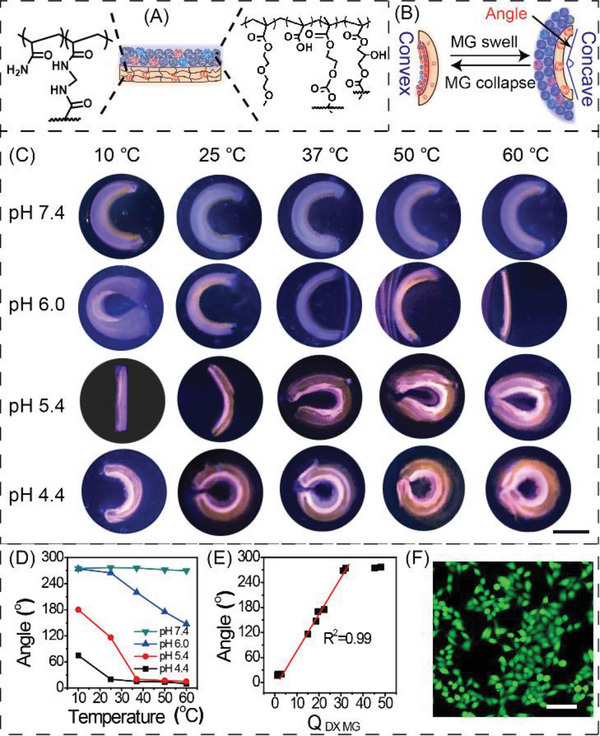
A) Schematic bilayer hydrogel actuator design. B) Depiction of changes of the MG gel layer causing actuation. C) Photographs of the bilayer gel at pH 4.4–7.4 as well as temperatures of 10–60 °C. The scale bar in (C) is 5.0 mm and applies to all images. D) Effects of temperature and pH on the bending angle. E) Variation of bending angle with volume swelling ratio (*Q*_DX MG_) of the gels. F) Live/dead assay for MCF‐7 cells in the presence of the actuator after 48 h. (F) The scale baris 100 µm.

Having established reversible pH‐, temperature‐, and cation‐responsive swelling and fluorescent color‐changing behavior (Figure S17, Supporting Information), we next constructed a versatile multiresponsive gel actuator by polymerizing DX MG‐CMA_0.07_/RDB_0.07_ atop a nonresponsive PAAm‐MG‐RDB_0.07_ gel (see **Figure** **5**A,B). Although PAAm‐MG‐RDB_0.07_ gels were swollen in the buffer solutions, the swelling ratio (*Q*_PAAm_) was independent of pH and temperature fluctuations (Figure S12, Supporting Information). At pH 7.4 the actuator presented as concave with angle‐invariant at all temperatures (Figure 5C, top row) due to stable and high *Q*_DX MG_ values for the DX MG gel layer (Figure S15A, Supporting Information). At pH 6.0 (Figure 5C, second row) the actuator changed from concave at 10 °C to flat at 50 °C because the *Q*_DX MG_ value decreased as temperature increased at this pH (Figure S15A, Supporting Information). At pH 5.4 the temperature‐dependent shape changes were most obvious (Figure 5C, third row) as the temperature‐induced change of *Q*_DX MG_ was greatest (Figure S15A, Supporting Information). At pH 4.4, the actuator was always convex with variation of bending angles (Figure 5C, bottom row). The change in the bending angle (*θ*) was measured to study the shape deformation of the actuators quantitatively (Figure 5B). As summarized in Figure 5D, the *θ* changed by 65°, 165°, 127°, and <7° at pH 4.4, 5.4, 6.0, and 7.4, respectively. Furthermore, the *θ* was proportional to *Q*_DX MG_ for *Q*_DX MG_ < 32 (Figure 5E), which is a potentially useful feature for designing actuators. These hydrogel actuators exhibit shape‐programming deformation intertwined with color‐shifting capabilities, akin to certain natural organisms capable of both morphological shifts and chromatic alterations. Live/Dead cell viability and MTS assays (Figure 5F; Figure S18, Supporting Information) were measured for MCF‐7 cells in the presence of the actuator. Because no cytotoxicity was detectable, our actuators and fluorescent DX MG gel have potential applications in bionic materials.

**Figure 6:**
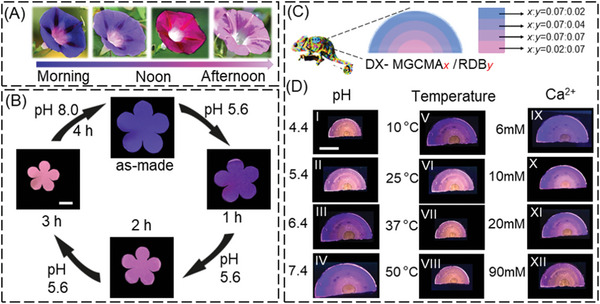
A) The color changes of true morning glory from morning to afternoon. B) Time‐dependent of color and size changes for artificial morning glory hydrogel in different buffer solution at 25 °C. The scale bar in (B) is 5.0 mm and applies to all images. C) Schematic design of artificial hydrogel skin of calotes versicolor. D) Photographs of the color changes of hydrogel skin with pH, temperature, and CaCl_2_ concentration variation. (D) The scale bar is 5.0 mm and applies to all images.

Many natural plants display captivating alterations in appearance, and the morning glory flower is a prime example of this phenomenon. It is known for its striking color changes, transitioning from blue in the morning to purple at midday, and gradually turning to red in the afternoon (**Figure** **6**A). These are caused by the presence of anthocyanins, which are abundant within the flower.^[^ ^33^ ^]^ When morning glory performs photosynthesis, it absorbs CO_2_ that reacts with H_2_O to generate H_2_CO_3_, consequently altering the pH and inducing the anthocyanins to turn pink.^[^ ^34^ ^]^ This intriguing behavior of the natural flower inspires us to fabricate artificial counterparts. As a proof‐of‐concept, a hydrogel flower film with a thickness of 1.0 mm having five petals was prepared using the DX MG‐CMA_0.07_/RDB_0.07_. To imitate the color of morning glory in the morning, we adjusted the pH value of MGs dispersion to 8.0 during synthesis. Hence, the as‐made artificial morning glory was initially blue. When it was placed in buffer solution (pH 5.6), the pH value of hydrogel matrix decreased gradually. As a result, our biomimetic morning glory hydrogel became purple after 1 h, fuchsia after 2 h, and finally pink after 3 h. More interestingly, the fluorescent hue could be reverted to blue by placing the hydrogel in a buffer solution of pH 8.0 (Figure 6B). The PL spectra were obtained and the calculated *I*_383_/*I*_581_ ratio decreased from 5.7 to 3.2 and recovered to 5.8 upon exposure to buffer solution of different pH value, respectively (Figure S19, Supporting Information). Our biomimetic hydrogel flower is like a real morning glory, with color changes synchronized with those of the natural flower in terms of color. It is well known that the pH values of water can decrease and increase when blubbing with CO_2_ and NH_3_, respectively. We can further develop the gas‐responsive hydrogel as a substantial water content within the hydrogel matrix. Thus, we believe that this ratio of MG‐CMA and MG‐RDB could effectively emulate the behavior of anthocyanins within flowers.

In addition to natural plants, many animals show variation in their skin color, body shape, or posture for camouflage or communication depending on environmental variables such as vegetation color, temperature, light, or mood.^[^ ^35^ ^]^ For instance, calotes versicolor are known for their exceptional color‐changing ability.^[^ ^36^ ^]^ They can morph their body into a plethora of hues, including pink, blue, red, orange, yellow, and even intricate combinations.^[^ ^37^ ^]^ The color‐changing mechanism is based on the alteration of the distance of guanine nanocrystals within iridophores on the surface of its dermis. In a calm state, the crystals are tightly packed, resulting in the reflection of only blue light, which combines with the yellow pigment to produce a green body coloration. When the chameleon is agitated, the nanocrystals are arranged actively in a looser structure. It reflects longer‐wavelength colors, such as red and yellow light, thereby yielding more vibrant structural colors.^[^ ^38^ ^]^ We have discussed and proved the distance changes of polymerized CMA and RDB in polymer network can lead to color variation of fluorescent DX MG, which results from the MG and DX MG swelling (or collapse) in response to multistimuli. Inspired by this mechanism, we attempted to mimic their intelligent skin in artificial systems using our newly developed multicolor fluorescent hydrogel. To this end, a multicolor fluorescent hydrogel was prepared by assembling the four layers of DX MG‐CMA*_x_*/RDB*_y_* with the ratio of *x* and *y* from 2:7 to 7:2 (Figure 6C). The artificial skin was complex colors and we used purple–pink–orange–orange to describe the multicolor from top to bottom layer. When it was immersed into a series of buffer solution from pH 4.4 to 7.4, the mixture color of multilayer hydrogel changed from light fuchsia–orange–orange–red (Figure 6D,I) to blue–purple–fuchsia–orange (Figure 6D,IV), accompanied by size increase. Similarly, the color of the entire artificial skin underwent a gradual chromatic shift from blue–purple–fuchsia–orange (Figure 6D,V) to fuchsia–orange–orange–red (Figure 6D,VIII), and the size decreased with elevating the temperature from 10 to 50 °C at pH 5.4. Another typical behavior of multicolor hydrogel is cation‐triggered synergistic change in color and size. When it was immersed in increased concentration of CaCl_2_ solution, the transition of color is more obvious, from blue (Figure 6D,IX) to fuchsia (Figure 6D,XII). Owing to reversibility of *Q*_DX MG_ and *I*_383_/*I*_581_ for the DX MG‐CMA_0.07_/RDB_0.07_ gels with pH changing, temperature switching and difference CaCl_2_ concentration at least for 4 cycles (Figure S17, Supporting Information), we believe the fluorescence behavior and the size of the bionic skin of calotes versicolor is reversible. In this way, our multicolor fluorescent hydrogel constructed by layer‐by‐layer technique to build intelligent skin is demonstrated for the first time, paving the road for accessible color‐changing biomimetic and soft material.

## 3 Conclusion

In this research, we have presented a simple and novel method for fabrication of biomimetic hydrogels by utilizing MGs with different fluorescent color (blue and orange). The MGs were synthesis involved the polymerization of fluorophore‐containing monomers. The fluorescent hydrogel was constructed via using OEG‐based vinyl functional MGs as building block and doping with two fluorescent MGs. This method effectively addresses several issues such as leakage of fluorophore and fluorescence deficiencies. The hydrogel is multiresponsive (pH, temperature, and cation) and showed synergistic changes in fluorescence color and size under multiple stimuli. Based on these results, we showcase the feasibility of integrating and optimizing size‐changing and multiemission‐color‐switching functionalities within a single hydrogel system triggered by multiple stimuli. As a proof of concept, we fabricated an actuator capable of controlled bending behavior and color visual display, modulated by adjustments in pH and temperature. We also demonstrated a morning glory‐inspired artificial hydrogel flower for the first time. Its color changing was dictated by the delicate interplay between different pH values of buffer solution. Furthermore, our hydrogel also emulates the color‐changing dynamics in the skin of calotes versicolor. This strategy offers a versatile platform for development of adaptable soft materials with color‐switching capabilities and holds significant potential applications in biomimetic soft robotics, biosensors, visual detection/display systems, and camouflage technology in future.

## 4 Experimental Section

### Materials

2‐(2‐methoxyethoxy)ethyl methacrylate (MEO_2_MA, 95%), MAA (98%), ethylene glycol dimethacrylate (98%), GMA (97%), sodium dodecyl sulfate (98.5%), ammonium persulfate (98%), K_2_CO_3_ (ACS grade), CHCl_3_ (≥99.5%), methanol (MeOH, ≥99.9%), methacryloyl chloride (97.0%), Rhodamine B, *N*‐*N*′‐methylene bisacrylamide (MBAAm, ≥99.5%), acrylamide (AAm, ≥99.5%), and 2,2′‐azobis(2‐methylpropionamidine) dihydrochloride (V‐50, 97%) were all purchased from Aldrich. 4‐Methylumbelliferone (≥98%), 2‐bromoethanol (95%), *N*,*N*‐dimethylformamide (ACS grade), ethanol (100%), triethylamine (99%), CH_2_Cl_2_ (≥99.8%), and Nile blue A were purchased from Fisher Scientific. Methacryloxyethyl thiocarbamoyl Rhodamine B (Me‐RDB) was purchased from Polysciences. All materials were used as received. Ultrahigh purity water was used that had been doubly filtered and deionized.

### Synthesis of MG‐CMA, MG‐RDB, and MG‐MEO Using Semicontinuous Precipitation Copolymerization

The synthesis of fluorescent MG (MG‐CMA and MG‐RDB) as well as nonfluorescent MG (MG‐MEO) were closely followed the procedure outlined in the previous report.^[^ ^39^ ^]^ All of them was synthesized via semicontinuous precipitation copolymerization. It is worth noting that the entire synthesis and storage process was conducted under protection from light, utilizing Al foil as a light‐blocking barrier. The detailed materials utilized in the preparation of the various MGs are presented in Table S1 of Supporting Information.

### Synthesis of GMG‐MEO via Vinyl‐Functionalizing MG‐MEO

The pH of the dialyzed MG‐MEO dispersion (1.0 wt%, 120 mL) was adjusted to 5.0 and GMA (0.65 mL) was added while stirring. The dispersion was stirred for 8 h at 40 °C. The obtained dispersion was washed with hexane twice and residual hexane removed through rotary evaporation to get the GMG‐MEO dispersion.

### Synthesis of Multiresponsive DX MG‐CMA*_x_*/RDB*_y_* Hydrogels

Doubly crosslinked microgel DX MG‐CMA*_x_*/RDB*_y_* gels containing preset amount of MG‐RDB and MG‐CMA were prepared. The *x* and *y* values represented the concentration of MG‐CMA and MG‐RDB (wt%) in mixture dispersion. For example, to constructed DX MG‐CMA_0.07_/RDB_0.07_, concentrated GMG‐MEO (2.205 mL, 15.0 wt%), MG‐CMA (192 µL, 1.0 wt%), MG‐RDB (192 µL, 1.0 wt%), and photoinitiator V50 (113.4 µL, 2.46 wt%) was mixed uniformly. The pH value was adjusted to 7.4 by adding aqueous NaOH solution (35 µL, 4.0 m). The solution was transferred to a rubber O‐ring (outer diameter = 13 mm and thickness = 1.8 mm), sandwiched between two quartz glass slides, sealed using clamps and polymerized at UV lamp of 365 nm for 5 min.

### Synthesis of Nonsensitive PAAm‐MGCMA*_x_*/RDB*_y_* Hydrogels

PAAm‐MGCMA*_x_*/RDB*_y_* hydrogels containing preset amount of MG‐RDB and MG‐CMA were prepared. The *x* and *y* values represented the concentration of MG‐CMA and MG‐RDB (wt%) in mixture dispersion. For example, to constructed AAm‐MGCMA_0.07_/RDB_0.07_, the comonomer solution (1.2 mL) containing AAm (20.4 wt%), MBAAm (0.3 wt%), MG‐CMA (0.07 wt%), MG‐RDB (0.07 wt%), and photoinitiator V50 (0.1 wt%) were mixed uniformly. The subsequent UV light‐induced polymerization procedure was executed in congruence with the methodology for the synthesis of DX MG‐CMA*_x_*/RDB*_y_* gels.

### Bilayer Hydrogel Actuator Preparation

The preparation procedure of bilayer hydrogel actuator was in accordance with the previous report.^[^ ^39^ ^]^ Herein, the first layer gel was PAAm‐MG‐RDB_0.07_ hydrogel and the second layer one was DX MG‐CMA_0.07_/RDB_0.07_. The precursor solution (1.09 mL) of first layer nonresponsive gel consisted of AAm (22.0 wt%), MBAAm (0.3 wt%), MG‐RDB (0.07 wt%), and photoinitiator V50 (0.1 wt%) and the mixture solution of second layer gel included GMG‐MEO (15.0 wt%, 2.205 mL), MG‐CMA (192 µL, 1.0 wt), MG‐RDB (192 µL, 1.0 wt%), NaOH (35 µL, 4.0 m), and V50 (113.4 µL, 2.46 wt%). The time of UV irradiation for both layers of gel is 5 min.

### Multicolor Hydrogels Preparation

The multicolor hydrogels were prepared by assembling the four layers of DX MG‐CMA*_x_*/RDB*_y_* with the ratio of *x* and *y* from 2:7 to 7:2. Mixture solution containing concentrated GMG‐MEO (15.0 wt%, 2.205 mL), MG‐CMA (55 µL, 1.0 wt%), MG‐RDB (192 µL, 1.0 wt%), H_2_O (137 µL), NaOH (35 µL, 4.0 m), and V50 (113.4 µL, 2.46 wt%) was transferred into the O‐ring (inter diameter = 5.0 mm and thickness = 1.0 mm) and polymerized by irradiation at 365 nm for 5 min to form the first layer gels. The O‐ring was taken and replaced by another (inter diameter = 8.0 mm and thickness = 1.0 mm). The mixture solution containing concentrated GMG‐MEO (15.0 wt%, 2.205 mL), MG‐CMA (192 µL, 1.0 wt%), MG‐RDB (192 µL, 1.0 wt%), NaOH (35 µL, 4.0 m), and V50 (113.4 µL, 2.46 wt%) was transferred into the free spaces into O‐ring and irradiation at 365 nm for 5 min to form the second layer gels. The third and fourth hydrogels were constructed by same procedure but change the bigger O‐ring (inter diameter = 11.0 mm and 14.0 mm; thickness = 1.0 mm) and the precursor solution with different ratio of MG‐CMA and MG‐RDB (7:4 and 7:2, respectively).

### Physical Measurements

UV–vis spectra were measured using a PerkinElmer Lambda 950 UV/vis spectrometer. PL spectra were obtained using Edinburgh Instruments FLS1000 spectrometer and the excitation wavelength was 302 nm. Titration measurements were conducted in present of aqueous NaCl (0.05 m) using a Mettler Toledo titration instrument. DLS data and electrophoretic mobility data were obtained using a Malvern Zetasizer NanoZS apparatus (Malven Instrument Ltd), fitted with a 20 mW HeNe laser and the angle of detection was set at 173°. TEM images were obtained using a TECNAI G2F30 instrument. Number‐average diameters from TEM images were obtained using ImageJ software. The volume swelling ratio values for the DX MG gels (*Q*_DXMG_) were determined gravimetrically. The DX MGs were placed into 0.10 m buffer solution and weighted every day for 5 days. The *Q*_DXMG_ values were calculated using Equation (1)

(1) $$Q_{\mathrm{DXMG}}=\rho_{p}\;\left(\frac{Q_{m}}{\rho_{s}}+\frac{1}{\rho_{p}}\right)-\frac{\rho_{p}}{\rho_{s}}$$

where *Q*_m_ is the ratio of swollen gel mass to the dry mass. The parameters ρ_s_ and ρ_p_ are the densities of water and the polymer (1.2 g mL^−1^), respectively.

### Cytotoxicity MTS Assays and Live/Dead Fluorescence Imaging

MCF‐7, human breast cancer cells, were cultured in Invitrogen α‐minimum essential medium (α‐MEM) supplemented with 5% fetal bovine serum (Thermo‐Fischer Scientific, USA), 1% sodium pyruvate (Gibco), 1% gluta MAXTM‐1 (Gibco), and 1% penicillin–streptomycin (Thermo‐Fischer Scientific, USA) at 37 °C and humidified 5% CO_2_ incubator. Cells were seeded at a density of 5 × 10^3^ cell in 24‐well plates (Costar Corning Corp.,) and cultured for 1 day. On the following days, the cells with updated 1.5 mL α‐MEM were exposed to 5 mm disks of gels (50 mg) for 1, 2, and 3 days. The gels were indirectly delivered to the adherent cells after being dispersed in α‐MEM. Viability of cells was determined at 24, 48, and 72 h time‐points by Live/Dead assay (Life Technologies, UK) and MTT assay (Sigma‐Aldrich) as per manufacturer's instructions. For the positive control, cells were incubated with pure α‐MEM. Images and absorbance were obtained with a confocal laser scanning microscopy (LSM 900 with Airyscan) and a microplate reader (Synergy H1, Bio‐Tek), respectively.

## Conflict of Interest

The authors declare no conflict of interest.

## Supporting information

Supporting Information

## Data Availability

The data that support the findings of this study are available from the corresponding author upon reasonable request.
