# Supplementary material for: Bioinspired Multistimuli‐Induced Synergistic Changes in Color and Shape of Hydrogel and Actuator Based on Fluorescent Microgels
Source: Adv Sci (Weinh). 2023 Nov 27;11(3):2304776. doi: 10.1002/advs.202304776 (PMC10797463; doi:10.1002/advs.202304776)
Supplement: Supplementary file 1 — Supporting Information [file ADVS-11-2304776-s001.pdf]

ADVANCED SCIENCE
Open Access

Supporting Information

for *Adv. Sci.*, DOI 10.1002/advs.202304776

Bioinspired Multistimuli-Induced Synergistic Changes in Color and Shape of Hydrogel and Actuator Based on Fluorescent Microgels

Dongdong Lu, Qing Lian and Mingning Zhu**

Supporting Information

Bioinspired Multistimuli-Induced Synergistic Changes in Color and Shape of Hydrogel and Actuator Based on Fluorescent Microgels

Dongdong Lu[a,b*], Qing Lian[b], Mingning Zhu[c*]

A. Dongdong Lu

School of Physical Sciences, Great Bay University, Dongguan 523808, P. R. China

E-mail: ludongdong@gbu.edu.cn

B. Dongdong Lu, Qing Lian

Derpartment of Materials Science and Engineering, Southern University of Science and Technology, Shenzhen 518055, P. R. China

C. Mingning Zhu

School of Biomedical Engineering, Guangdong Medical University, Dongguan 523808, P. R. China

E-mail: zhumn@gdmu.edu.cn

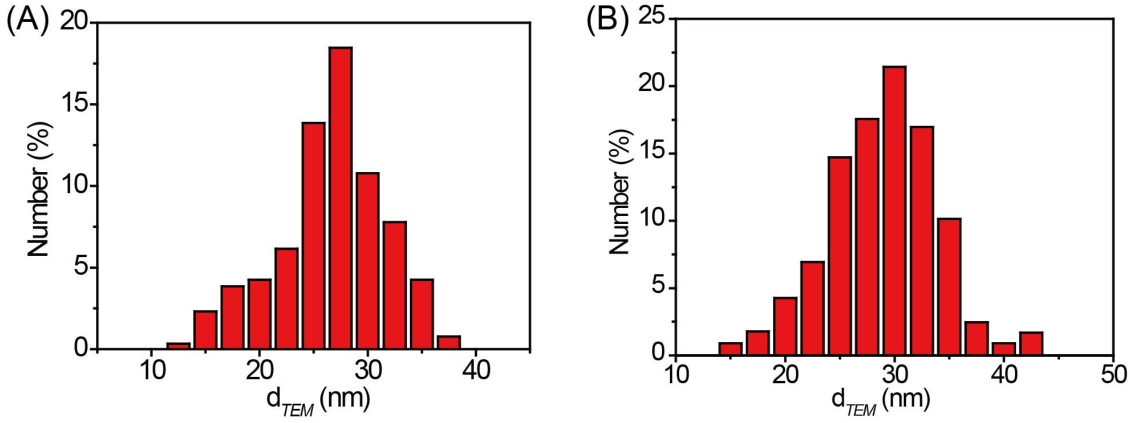

Figure S1. The size distributions of TEM for MG-CMA **(A)** and MG-RDB **(B)**.

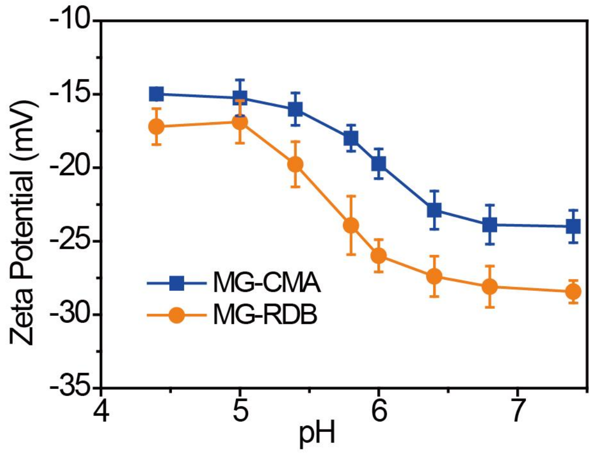

Figure S2. Zeta potential (ζ) with pH measured for two MG dispersions.

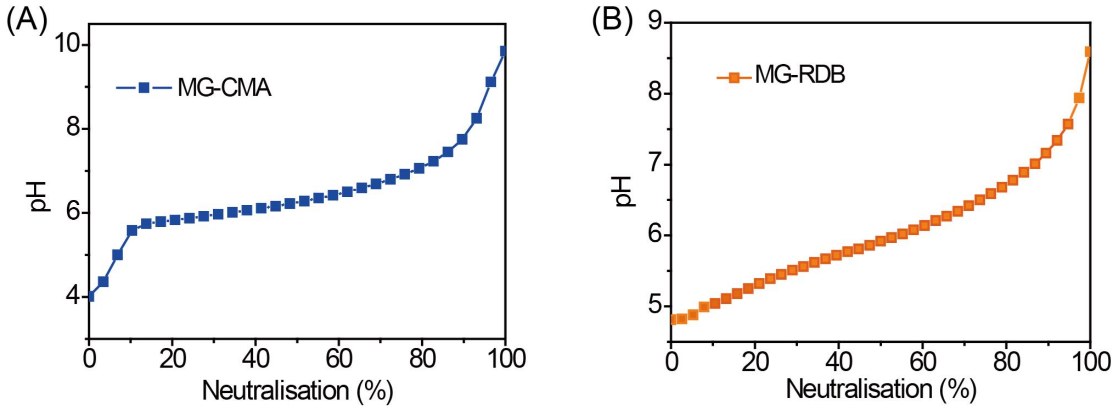

Figure S3. Potentiometric titration data for the two MG dispersions.

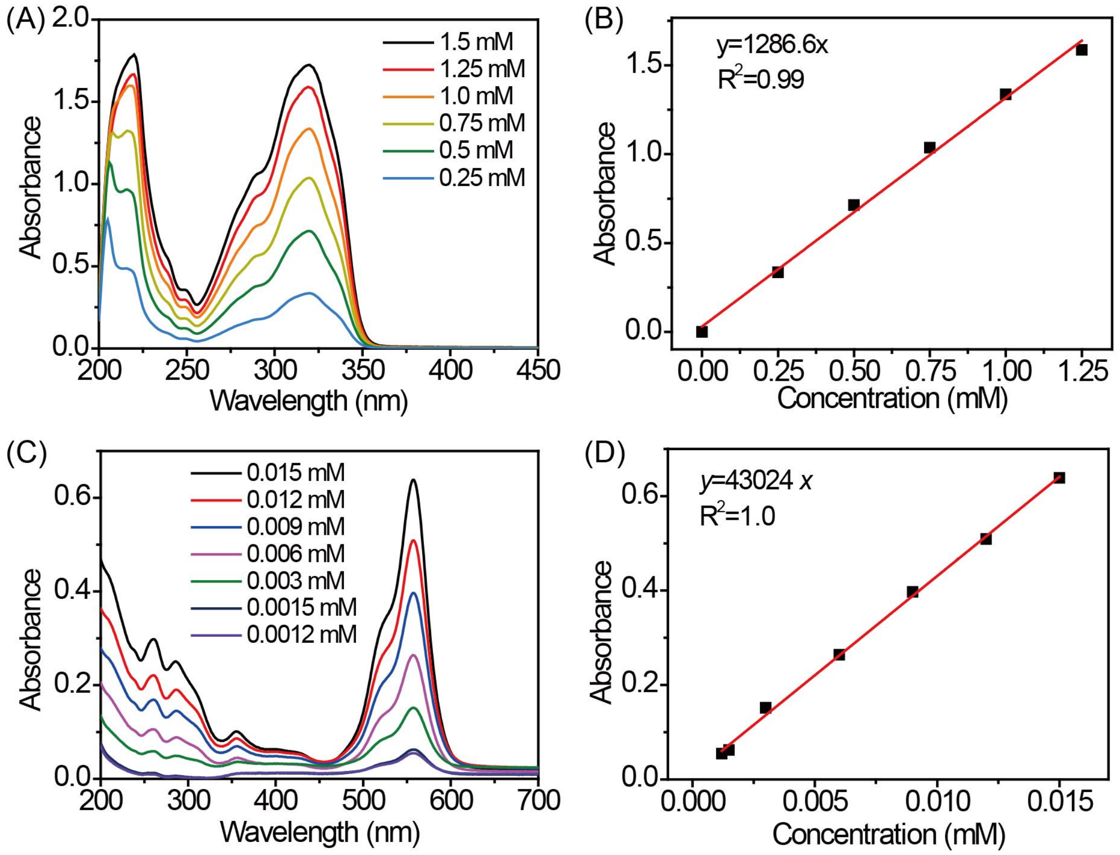

Figure S4. UV-visible spectra for CMA at various concentrations in methanol **(A)** and RDB in H_2O **(C)**. Variation of absorbance at 320 nm with CMA concentration **(B)** and 557 nm with RDB concentration **(D)**. The molar extinction coefficient was calculated from (B) and (D) as 1286.6 and 43024 $mol^{-1}\ dm^3\ cm^{-1}$, respectively.

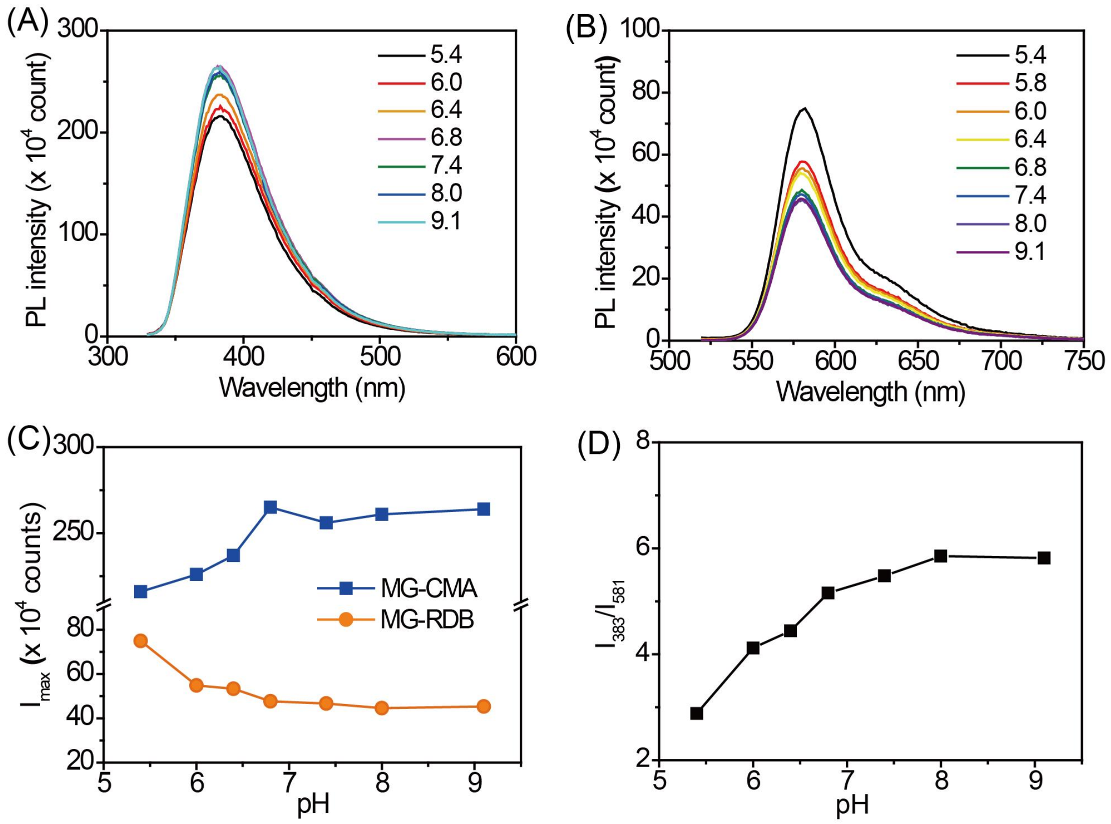

Figure S5. PL spectra for MG-CMA **(A)** and MG-RDB **(B)** with different pH values. PL maximum intensity (I_{max}) **(C)** and the change of I_{383}/I_{581} ratio **(D)** for MG-CMA and MG-RDB**.** The data was obtained at 25 °C.

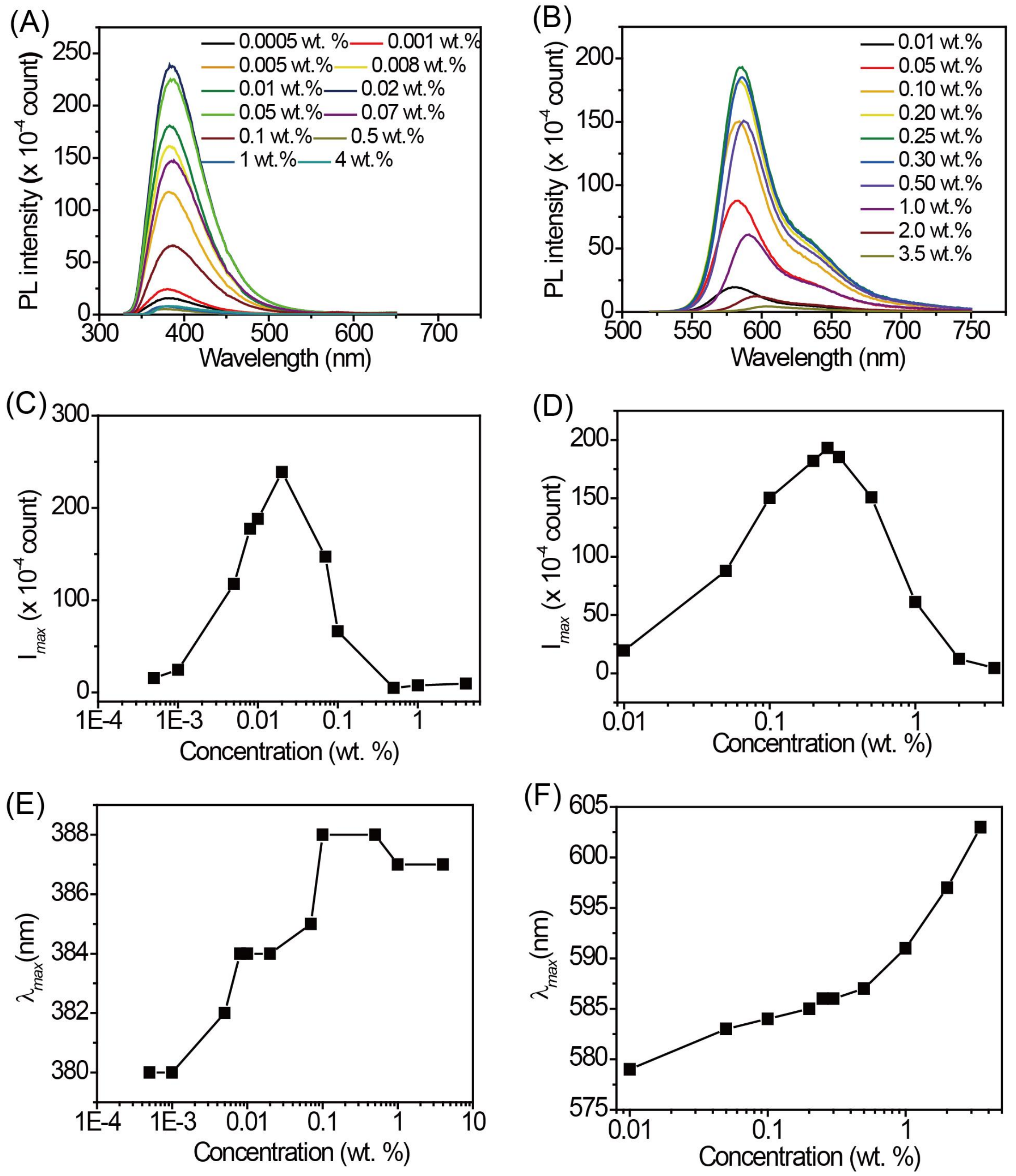

Figure S6. Concentration dependence of MG-CMA in PL spectra **(A)**, $I_{\max}$ **(B)** and $\lambda_{\max}$ **(C)**. Concentration dependence of MG-RDB in PL spectra **(D)**, $I_{\max}$ **(E)** and $\lambda_{\max}$ **(F)**. The data was obtained at 25 °C.

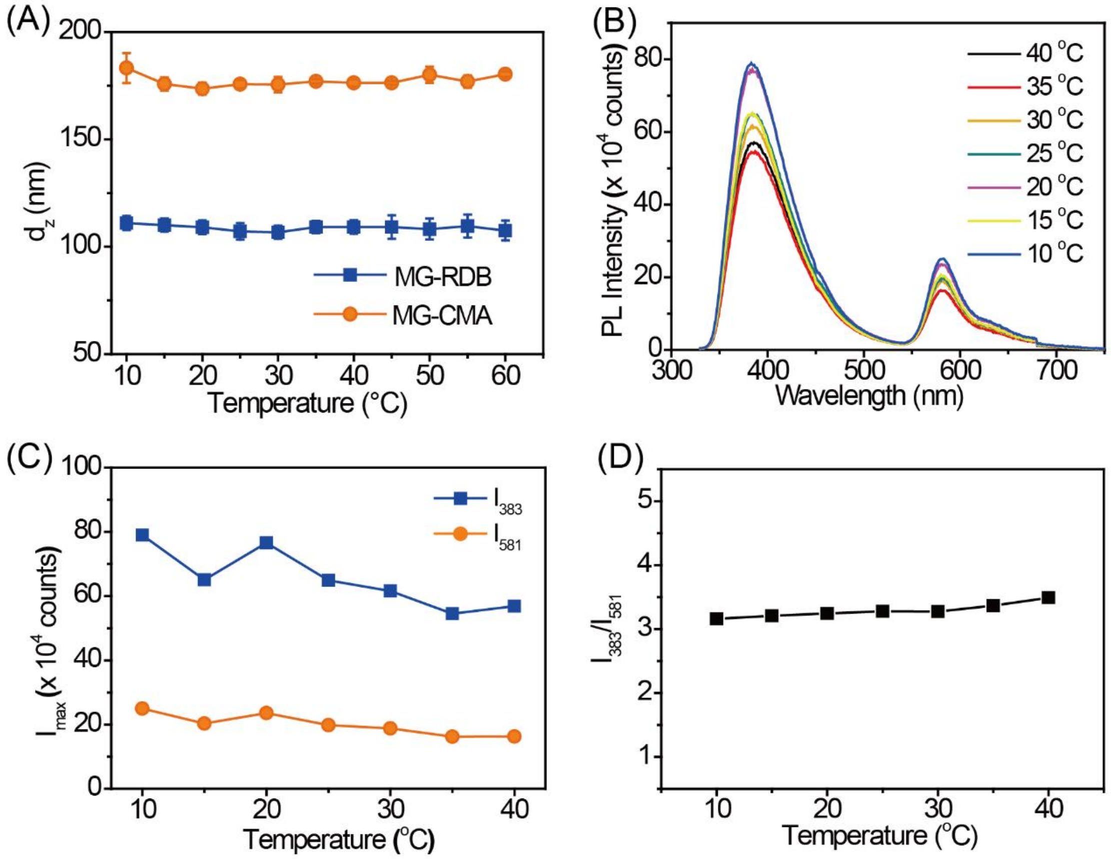

Figure S7. **(A)** Variation of d_z with different temperature at pH 7.4 measured for MG dispersions. PL spectra **(B)**, I_{max} **(C)** and change of I_{383}/I_{581} ratio **(D)** for mixture dispersion of MG-CMA and MG-RDB with different temperature at pH 7.4. The concentration of MG-CMA and MG-RDB are 0.07 wt.%.

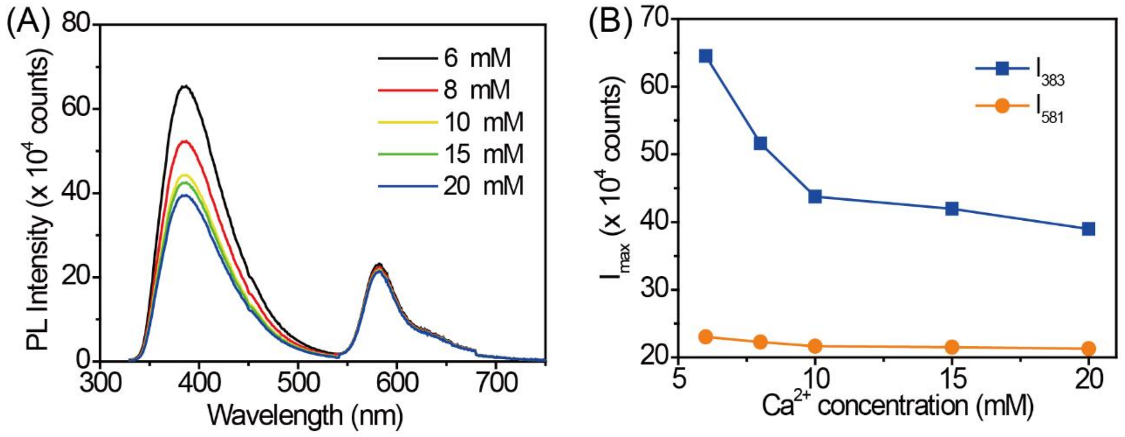

Figure S8. PL spectra **(A)** and I_{max} **(B)** for mixture dispersion of MG-CMA and MG-RDB with $CaCl_2$ concentration at pH 7.4. The concentration of MG-CMA and MG-RDB are 0.07 wt.%. The data was obtained at 25 °C.

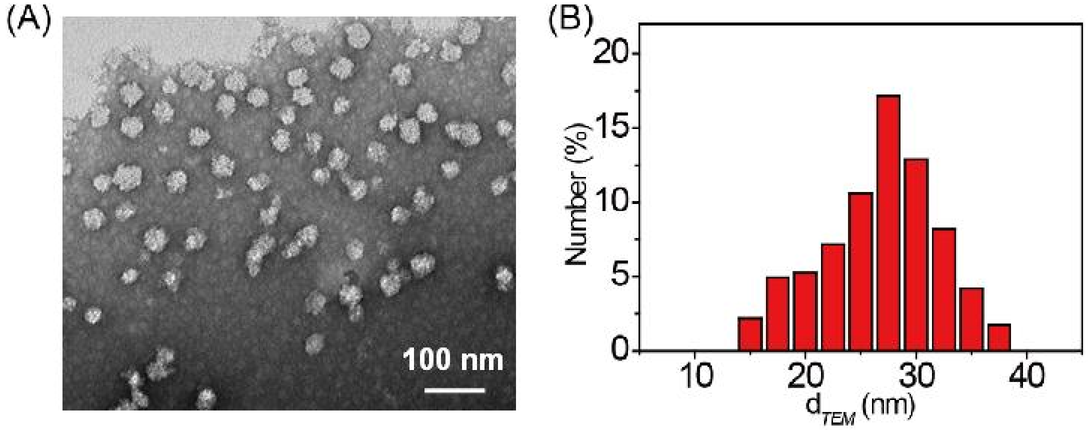

Figure S9. TEM image **(A)** and the size distributions of TEM **(B)** for MG-CMA and MG-RDB mixture dispersion.

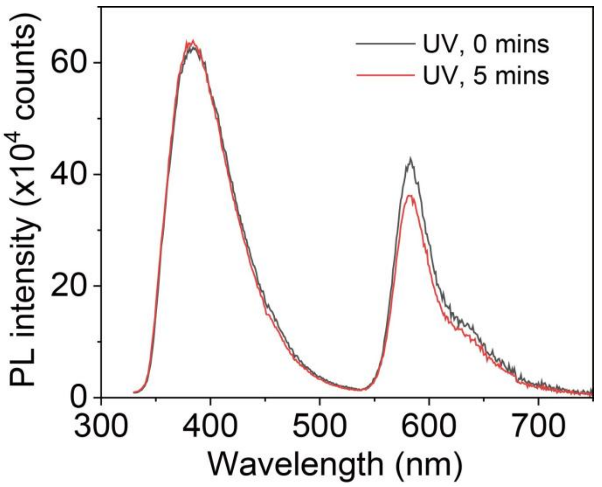

Figure S10. PL spectra of mixture dispersion of GMG-MEO, MG-CMA and MG-RDB (UV, 0 mins) and DX MG-CMA$_{0.07}$/RDB$_{0.07}$ (UV, 5mins). It indicated the UV light (365 nm) has a negligible effect on the fluorescence behaviour..

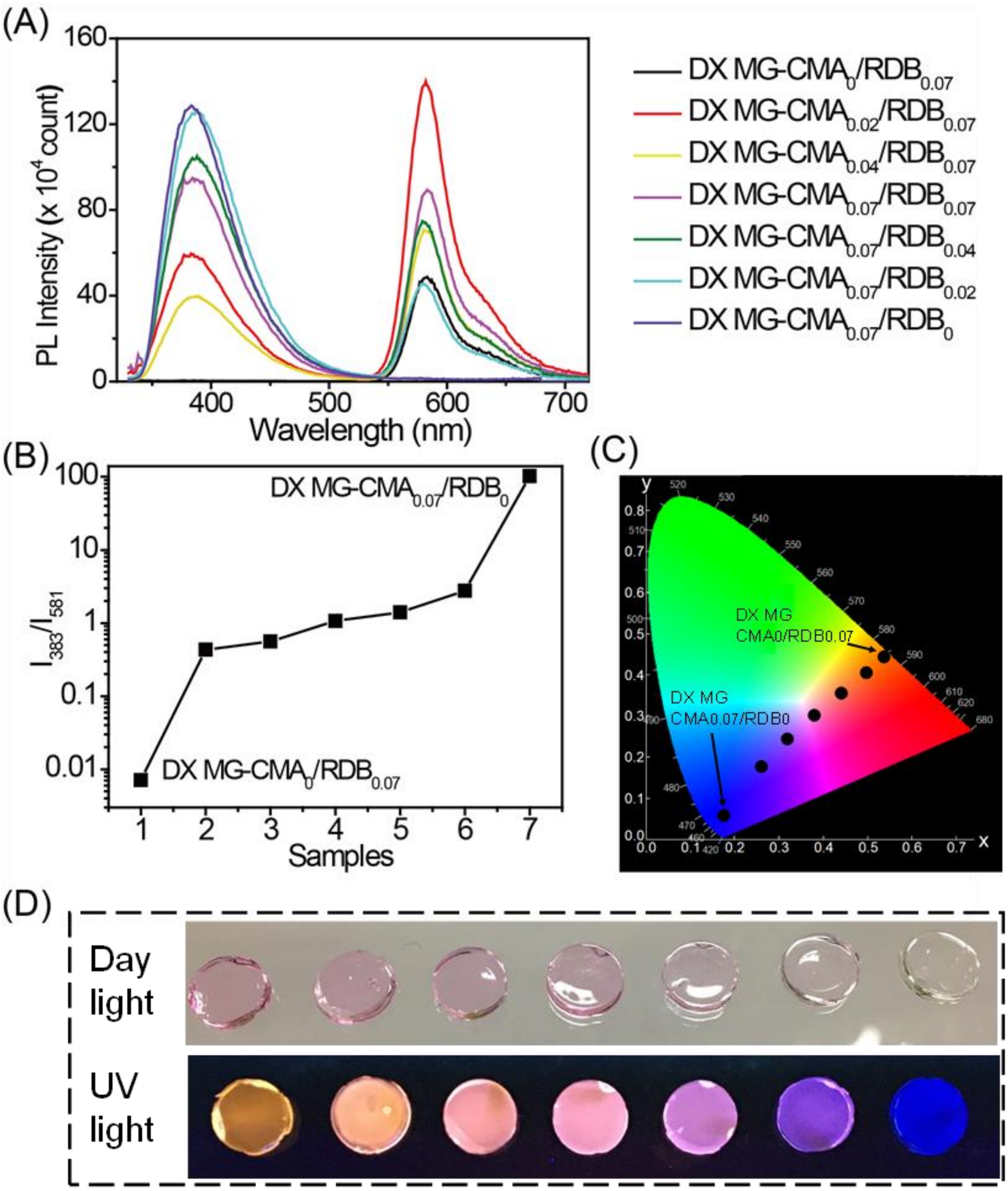

Figure S11. PL spectra **(A)**, change of I_{383}/I_{581} ratio **(B)**, CIE diagram **(C)** of multicolor fluorescent DX MG-CMA$_x$/RDB$_y$ hydrogels, which are prepared by different ratio of MG-CMA and MG-RDB. **(D)** the photos of DX MG-CMA$_x$/RDB$_y$ hydrogels under daylight and UV lamp with 302 nm. The hydrogels are DX MG-CMA$_x$/RDB$_y$ with *x* value increase and *y* value decrease from left to right. The data was obtained at 25 °C.

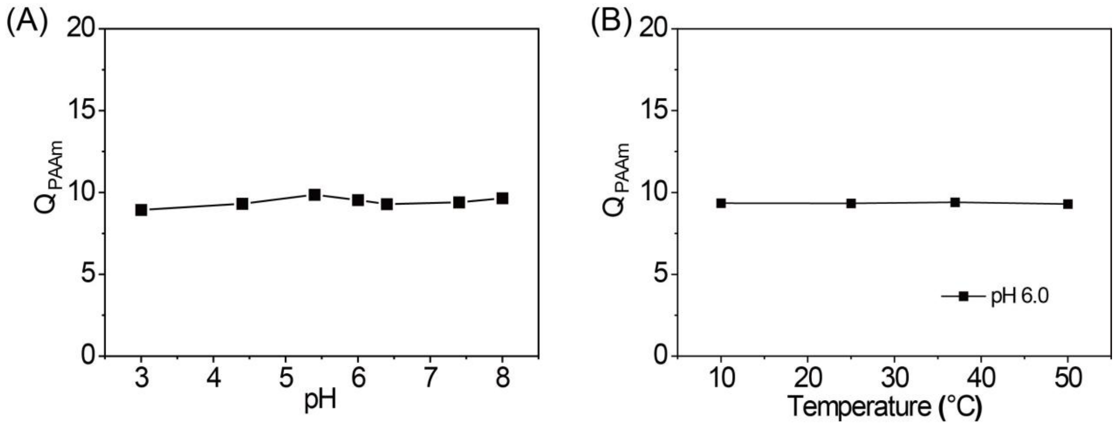

Figure S12. Variation of Q_{PAAm} with pH (A) and temperature (B) measurement of PAAm-MG-RDB$_{0.07}$ gels. The data in (A) was obtained at 25 °C.

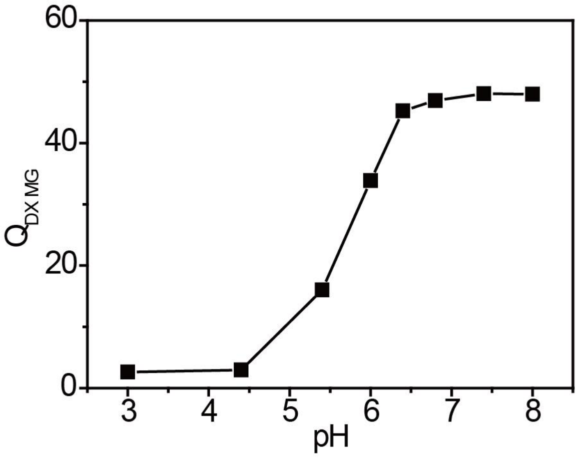

Figure S13. Variation of $Q_{DX\ MG}$ with pH measurement of DX MG-CMA$_{0.07}$/RDB$_{0.07}$ hydrogels. The data was obtained at 25 °C.

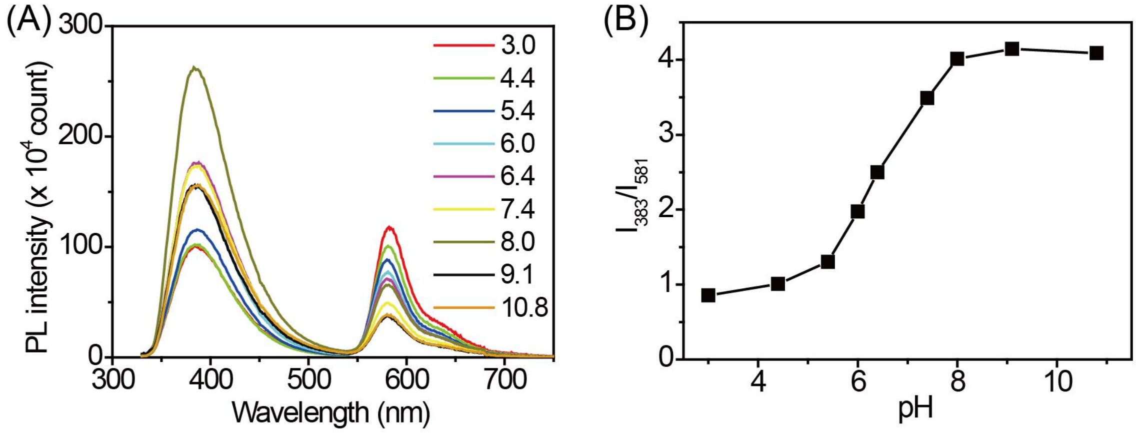

Figure S14. PL spectra (**A**), change of I_{383}/I_{581} ratio (**B**) with pH measurement of PAAm-$CMA_{0.07}$/$RDB_{0.07}$ hydrogels. The data was obtained at 25 °C.

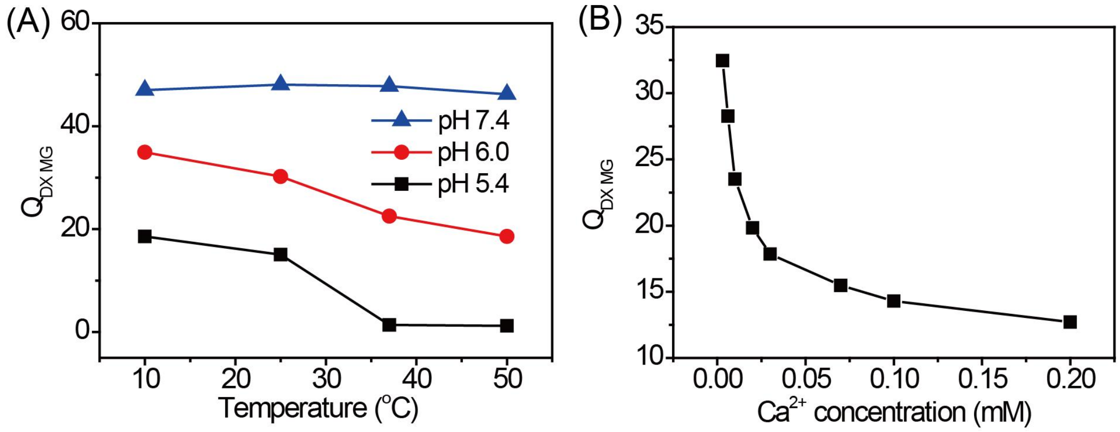

Figure S15. Variation of $Q_{DX\ MG}$ with different temperature at pH 5.4, pH 6.0 and pH 7.4 **(A)** and different $CaCl_2$ concentration measurement **(B)** of DX MG-$CMA_{0.07}$/$RDB_{0.07}$ hydrogels. The experimental condition of (B) is pH 7.4 at 25 ℃.

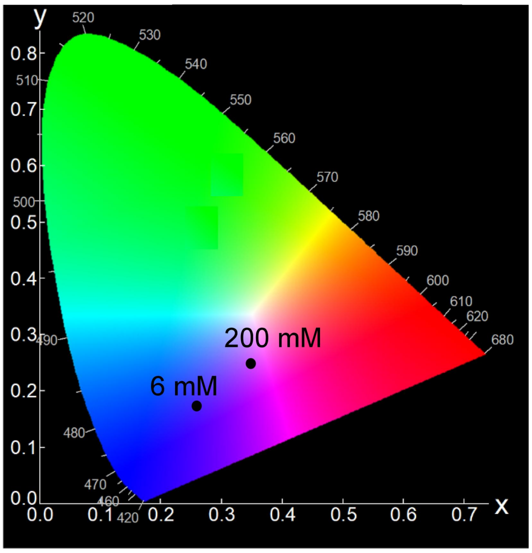

Figure S16. The CIE diagram of DX MG-$CMA_{0.07}$/$RDB_{0.07}$ hydrogels immersed in the solutions of different $CaCl_2$ concentration.

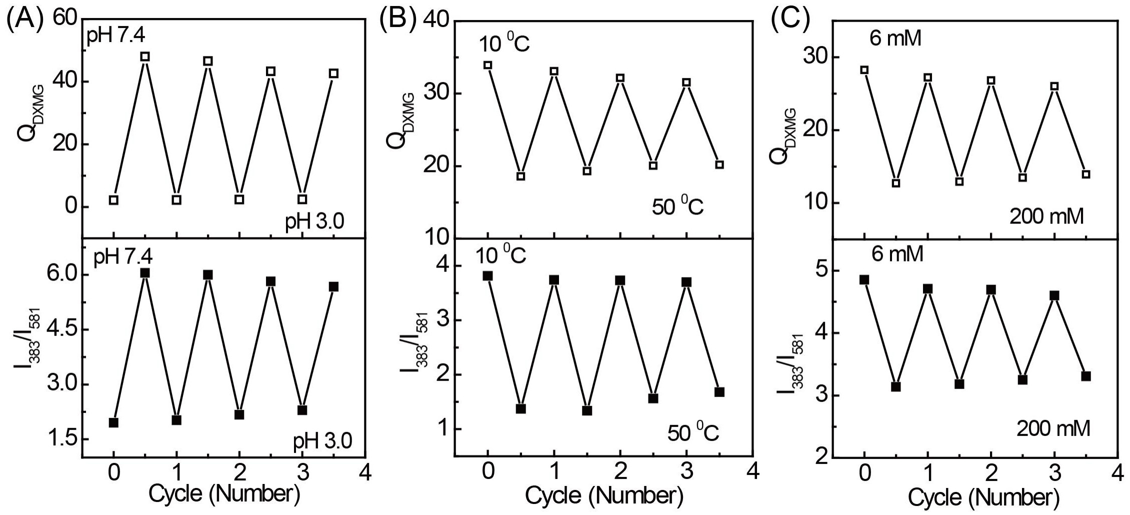

Figure S17. Reversibility of $Q_{DX\ MG}$ and I_{383}/I_{581} for the DX MG-$CMA_{0.07}$/$RDB_{0.07}$ gels due to pH changing **(A)**, temperature switching at pH 6.0 **(B)** and difference $CaCl_2$ concentration at pH 7.4 **(C)**. The temperature of experiment in (A) and (C) is 25 °C.

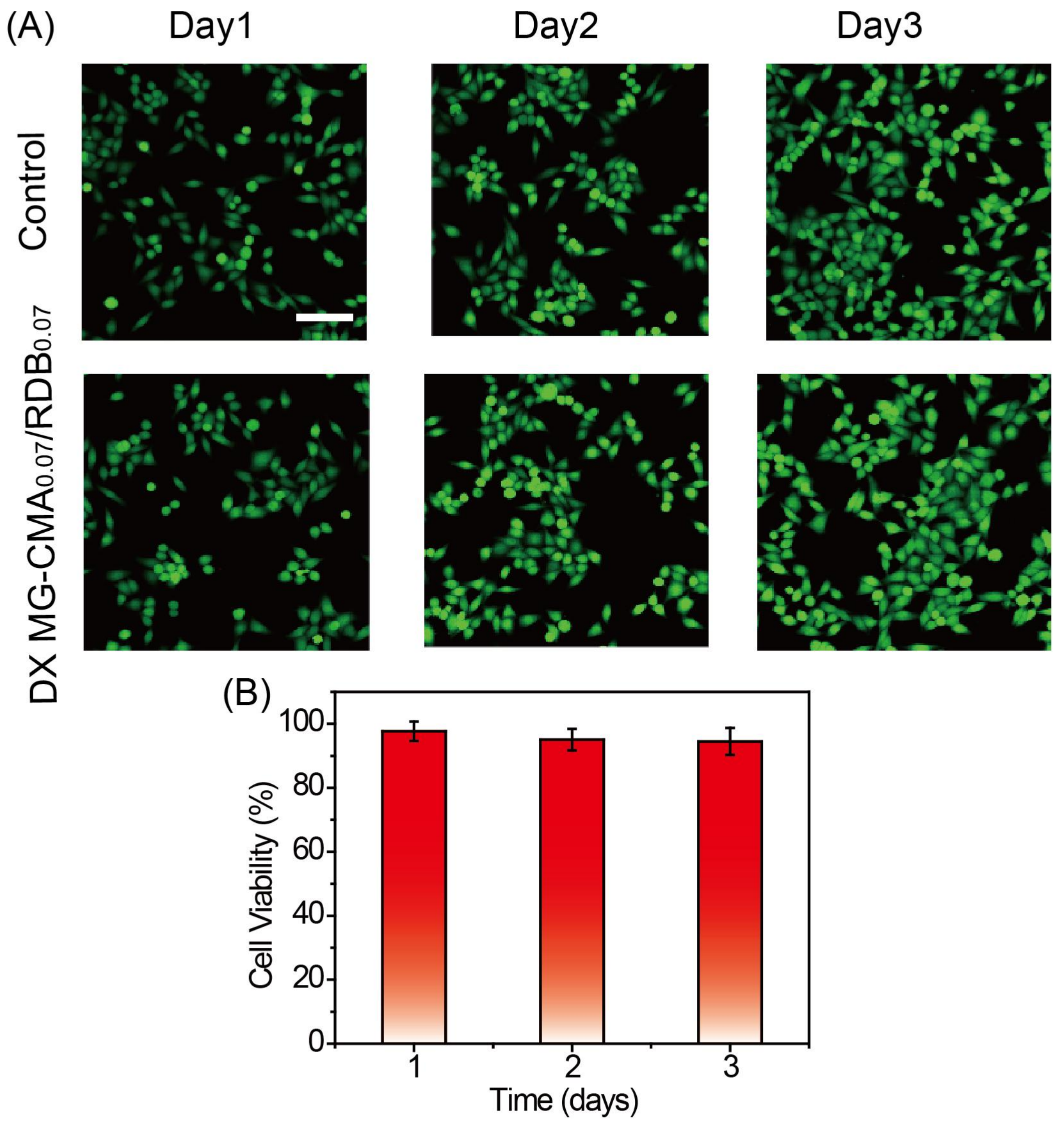

Figure S18. **(A)** Live/Dead cell assay and **(B)** cell viability calculated from MTT assay for the bilayer gel used to construct the actuators in Figure 5. MCF-7 cells were used. The control is the cells in the absence of the gel. The scale bar in (A) is 100 μm.

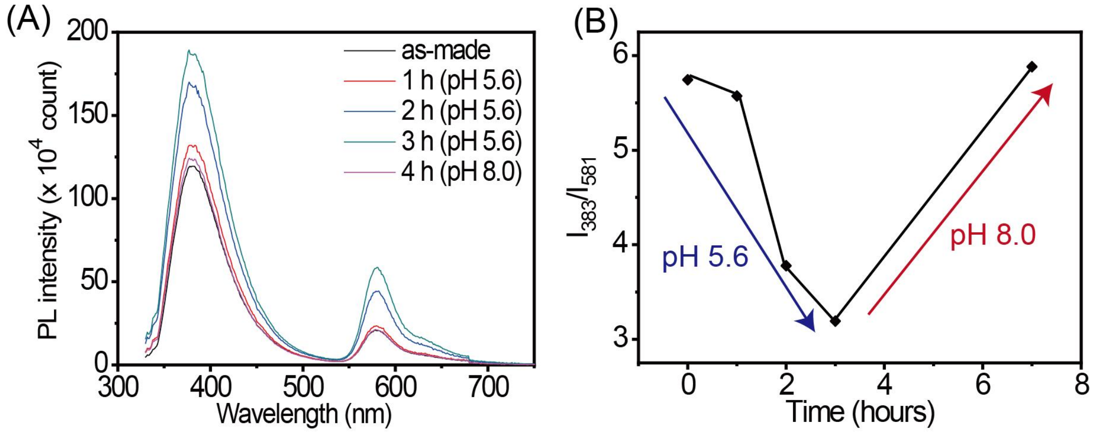

Figure S19. PL spectra **(A)**, change of I_{383}/I_{581} ratio **(B)** of biomimetic morning glory hydrogel when exposed with buffer solution of different pH values.

Table S1. Materials used to prepare the MGs

MGs	MEO_2MA /wt. % [a]	MAA /wt. % [a]	CMA /wt. % [a]	RDB /wt. % [a]	EGD /wt. % [a]	SDS /wt. % [b]	APS /wt. % [b]	Monomer mass / g [c]	H_2O / g
MG-RDB	82.3	14.5	-	1.9	1.2	0.55	0.05	2.1	20
MG-CMA	73.6	12.0	13.7	-	-	0.55	0.05	3.0	40
MG-MEO	83.5	15.0	-	-	1.5	0.55	0.05	6.2	60

[a]With respect to monomer. [b]Dissolved in water phase. [c]Total monomer was added during polymerization.

Table S2. Composition and properties of the MGs studied

MGs	MEO_2MA /mol%	MAA /mol% [a]	CMA /mol%[b]	RDB /mol%[b]	EGD /mol%[c]	GMA /mol%[d]	pK_a[e]	d_{TEM} /nm[f]	d_z/nm[g] (pH 5.4, 60°C)
MG-RDB	72.1	27.7	-	0.25	1.0	-	6.0	37 (8)	48 (0.07)
MG-CMA	66.9	26.7	6.35	-	-	-	6.1	30 (7)	40 (0.05)
GMG-MEO	64.9	28.2	-	-	1.0	5.9	6.0	41(7)	58(0.06)

[a]Determined from potentiometric titration data. [b]Determined from UV-visible spectroscopy data using the Beer Lambert law (Figure S4). [c]Based on the crosskiner used during polymerization. [d] Calculated using the difference of the MAA content before and after functionalisation. [e]Apparent pK_a value determined from potentiometric titration data. [f]The numbers in brackets are the standard deviation.[g]The numbers in brackets are the PDI values
